# The Function of the Lysophospholipase 1 (*LYPLA1*) Gene in Fetal Sheep Myoblast Proliferation and Myogenic Differentiation

**DOI:** 10.3390/ani15233474

**Published:** 2025-12-02

**Authors:** Zhanpeng Zhang, Yi Yin, Huiguo Yang, Kanati Shalike, Yanglin Nie, Xiukai Cao, Zehu Yuan, Wei Sun

**Affiliations:** 1 Joint International Research Laboratory of Agriculture and Agri-Product Safety of Ministry of Education of China, Yangzhou University, Yangzhou 225009, China; 13401726427@163.com (Z.Z.); 15995121804@163.com (Y.Y.); cxkai0909@163.com (X.C.); yuanzehu@yzu.edu.cn (Z.Y.); 2 International Joint Research Laboratory in Universities of Jiangsu Province of China for Domestic Animal Germplasm Resources and Genetic Improvement, Yangzhou University, Yangzhou 225009, China; 3 College of Animal Science and Technology, Yangzhou University, Yangzhou 225009, China; 4 Institute of Animal Husbandry, Xinjiang Academy of Animal Sciences, Urumqi 830013, China; yanghuiguo3239@163.com (H.Y.); kanati24239@sina.com (K.S.); 5 Anin Dairy (Jiangsu) Co., Ltd., Huaian 211600, China; compus123@163.com

**Keywords:** sheep growth, *LYPLA1*, myoblasts, proliferation, differentiation

## Abstract

Evidence from genome-wide association studies (GWAS) links genetic variants near the *LYPLA1* locus to variations in sheep muscle development. To further investigate whether *LYPLA1* expression plays a role in fetal sheep myoblast development, we performed gain-of-function/loss-of-function analyses targeting *LYPLA1* using siRNA and recombinant vectors. CCK-8 assays, EdU staining, and flow cytometry were used to investigate the effects of both *LYPLA1* knockdown and overexpression on the proliferation of fetal sheep myoblasts. Furthermore, this study investigated the effect of *LYPLA1* on myogenic differentiation by means of indirect immunofluorescence staining with MyHC and quantitative analysis of myogenic regulatory factor (MRF) expression. The results showed that downregulation of *LYPLA1* significantly reduced cell viability and proliferation rate, arrested myoblasts in the G2 phase, and inhibited proliferation. These effects were reversed when *LYPLA1* was overexpressed. Furthermore, *LYPLA1* knockdown significantly impaired myotube formation during terminal differentiation (days 5–7). This defect was correlated with a marked reduction in the expression of myogenic regulatory factors (MRFs). This effect was reversed when *LYPLA1* was highly expressed. In summary, this study demonstrates that *LYPLA1* promotes the proliferation and differentiation of fetal sheep myoblasts, establishing its potential as a target for the molecular breeding of sheep.

## Simple Summary

This study aimed to investigate whether a candidate gene (*LYPLA1*) associated with sheep growth affects the proliferation and differentiation of fetal sheep myoblasts. Myoblasts were isolated from fetal sheep skeletal muscle. Our findings revealed that knocking down *LYPLA1* gene activity significantly reduced muscle cell proliferation and differentiation. Conversely, increasing *LYPLA1* gene activity effectively promoted myoblast proliferation and differentiation. These results demonstrate that *LYPLA1* influences fetal sheep myoblast proliferation and differentiation. This study provides important theoretical support for the use of *LYPLA1* as a molecular marker for improving sheep meat traits and is expected to have potential value in improving livestock production efficiency and promoting agricultural economic development.

## 1. Introduction

Sheep are widely raised due to their strong adaptability to the environment and high reproductive capacity, thus holding significant importance in the agricultural economy. Sheep growth traits are one of the main economic traits. Maximizing the economic value of sheep during the breeding process is also the main goal of breeders [1]. Therefore, the genetic improvement of sheep growth traits, especially research on muscle growth and development, has been widely studied.

In recent years, genome-wide association studies (GWAS) have provided important clues for the genetic improvement of sheep by identifying genetic variants associated with sheep growth [2,3,4,5,6]. In a GWAS study on milk production traits in 1813 ewes [7], the candidate gene *LYPLA1* was found to be associated with milk production. In addition, WANG et al. sorted and analyzed the whole-genome resequencing genotypes from 1150 sheep and found that the *LYPLA1* gene may significantly affect the weight of sheep [8]. Studies have found that the protein encoded by the *LYPLA1* gene has hydrolysis S-palmitoylation modification and participates in the regulation of Wnt/β-catenin, Ras, and other signaling pathways [9]. Protein palmitoylation is a potent post-translational modification and is the only reversible lipid modification identified to date [10]. Current proteomic analyses have identified nearly a thousand palmitoylated proteins, which have been observed to be involved in specific biological mechanisms, such as differentiation, activation, immune response, proliferation, and migration [11,12], and play important functions in organisms. In view of the currently known GWAS results and the function of the *LYPLA1* gene, we speculate that the *LYPLA1* gene plays an important role in the muscle growth and development of sheep as a potential candidate gene.

The basic process of skeletal muscle development begins in early embryonic development, originating from mesenchymal stem cells in the mesoderm [13]. Mesenchymal stem cells form myogenic progenitor cells under the regulation of transcription factors and further differentiate into myoblasts. The withdrawal of myoblasts from the cell cycle after proliferation cessation is followed by their differentiation and fusion into multinucleated myotubes [14,15,16]. Existing research shows that the amount and quality of meat produced by animals are closely related to the number of muscle fibers, and the number of muscle fibers of animals is determined during pregnancy; that is, the number of muscle fibers will no longer increase after delivery [17,18]. Therefore, the prenatal period is a key factor in determining the quality and composition of an individual’s skeletal muscle. This stage not only determines the number of muscle fibers and muscle quality after birth but also has a profound impact on its subsequent production efficiency [19,20,21]. Therefore, this study directly targets the fetal development stage, using a sheep fetal myoblast model to analyze key cellular events in muscle fiber formation and establish a theoretical basis for improving mutton yield and quality from the developmental source. Since there has been no research on the effect of the *LYPLA1* gene on the function of sheep myoblasts, we selected fetal sheep myoblasts as experimental materials to verify the function of the *LYPLA1* gene.

In this study, we sought to elucidate the effects of the *LYPLA1* gene on the proliferation and differentiation of fetal sheep myoblasts by performing functional analyses at the cellular level. First, we quantified the expression levels of myogenic regulatory factors (MRFs) in fetal sheep myoblasts at different differentiation stages and determined that the isolated and purified fetal sheep myoblasts had good differentiation potential. Subsequently, we constructed an *LYPLA1* gene overexpression vector and siRNA to study the effect of the *LYPLA1* gene on myoblast proliferation. Finally, this experiment established an in vitro myoblast differentiation model to study the effect of the *LYPLA1* gene on myoblast differentiation. In summary, this study hopes to clarify the regulatory role of the *LYPLA1* gene in sheep muscle development at the cellular level through the above experiments in order to provide a theoretical basis for the genetic improvement of muscle growth in sheep.

## 2. Materials and Methods

### 2.1. Ethical Statement

The animal experiments in the current study were reviewed and approved by the Experimental Animal Ethics Committee of Yangzhou University (approval number 202103279, approval date 8 March 2021).

### 2.2. Sample Collection

The Hu sheep used in this study were supplied by Suzhou Taihu Dongshan Sheep Industry Development Co., Ltd. (Suzhou, China). In this experiment, we selected two healthy adult pregnant ewes of similar weight (the gestation age of their fetuses was approximately 85 days) and collected longissimus dorsi muscle tissue samples from the two fetuses for myoblast isolation. To ensure the sterility and integrity of the samples, we used strictly sterilized equipment to collect tissue samples such as the longissimus dorsi muscle, heart, lung, kidney, and liver tissue from Hu sheep. Following collection, tissue samples were immediately frozen in liquid nitrogen. All samples were subsequently stored at −80 °C in the laboratory prior to subsequent experiments.

### 2.3. Isolation, Purification, and Identification of Fetal Sheep Myoblasts

Primary sheep myoblasts were isolated from sheep longissimus dorsi muscle tissue via collagenase type II digestion [22]. The cell culture incubator was set at 37 °C and 5% CO_2_, and the cells were cultured in high-glucose DMEM medium (complete medium) containing a mixture of 10% fetal bovine serum (FBS) and 2% penicillin–streptomycin. The cells were purified via differential adhesion using a previous method [23]. After purification, primary myoblasts were obtained, which were then digested with 0.25% trypsin and transferred into 1.5 mL cryovials. They were then stored in liquid nitrogen for long-term use in subsequent experiments.

### 2.4. Vector Construction and Small Interfering RNA Synthesis

The small interfering RNA used in this experiment was designed and synthesized by GenePharma Pharmaceutical Technology Co., Ltd. (Shanghai, China). The specific sequence information of the small interfering RNA (siRNA) targeting the sheep *LYPLA1* gene is shown in Table 1. Among them, NC (negative control) was used as a negative control.

The *LYPLA1* gene was amplified via PCR using complementary DNA (cDNA) extracted from sheep tissue as a template. The designed primers are listed in Table 2. In this experiment, we used the pcDNA3.1(+) vector, which does not contain the fluorescent protein gene. The recombinant vector was constructed according to the instructions of the ClonEx-press II One-Step Cloning Kit (Vazyme Biotech Co., Ltd., Nanjing, China). The sequenced vector was named pcDNA3.1(+)-*LYPLA1* and stored at −20 °C for subsequent experiments.

### 2.5. Cell Transfection and Induced Differentiation

According to the instructions of jetPRIME transfection reagent (Polyplus transfection, Strasbourg, Illkirch, France), when the confluence of the myoblasts after passage reached 40–50%, transfection with small interfering RNA fragments targeting the *LYPLA1* gene was performed; when the cell confluence reached 60–70%, transfection with an *LYPLA1* overexpression recombinant plasmid was performed. The transfection groups were set up as follows: for overexpression, pcDNA3.1(+) and pcDNA3.1(+)-*LYPLA1*; for knockdown, siRNA-*LYPLA1* and NC. Each group was assayed in three replicates. When the confluence reached 80–90% post-transfection, the complete growth medium (GM, 90% high-glucose DMEM, 10% FBS) was replaced with differentiation medium (DM, 98% high-glucose DMEM, 2% horse serum), which was changed every 48 h, to induce in vitro differentiation of fetal myoblasts.

### 2.6. Total RNA Extraction and Real-Time Quantitative Polymerase Chain Reaction (RT-qPCR)

Total RNA extraction from all samples in this experiment was performed according to the TRNzol Universal Total RNA Extraction Kit (TIANGEN, Beijing, China). The extracted RNA was detected and then stored at −80 °C. We performed reverse transcription using the FastKing One-Step Genomic cDNA First-Strand Synthesis Premix (TIANGEN, Beijing, China) according to the manufacturer’s instructions, and the reaction system was as follows: 4 μL of 5× FastKing-RT SuperMix, 1 μg of Total RNA, and RNase-Free ddH2O to 20 μL. The reaction program was set as follows: 42 °C for 15 min, 95 °C for 3 min, and a stop temperature of 4 °C. The RT-PCR reaction reagent used was ChamQ SYBR qPCR Master Mix (Vazyme Biotech Co., Ltd., Nanjing, China). Cell proliferation markers *CDK2*, *Cyclin D1*, and *PCNA* were selected to reflect G1/S phase transition, early process, and DNA replication activity, respectively, so as to comprehensively judge the proliferation level. The myogenic differentiation markers *MyoD1*, *Myf5*, *MyoG*, and *MRF4* were selected to span the entire myogenesis process, from lineage commitment (*MyoD1*/*Myf5*) through terminal differentiation (*MyoG*) to functional maturation (*MRF4*). Primers were designed using the Primer-BLAST tool on the NCBI website. The primers are listed in Table 3.

### 2.7. 5-Ethynyl-2′-deoxyuridine (EdU) Assay for Cell Proliferation

Subsequent EdU staining experiments were performed according to the Cell-Light EdU Apollo In Vitro Kit protocol (RIBOBIO, Guangzhou, China). First, myoblasts were plated in 96-well plates with GM. After transfection, EdU staining and Hoechst staining were performed when the cell density reached approximately 90%. Second, fluorescence images were taken using an inverted fluorescence microscope (Nikon, Minato, Tokyo, Japan). Finally, we quantified the cell number in each field using Image J (National Institutes of Health, Bethesda, MD, USA). The cell proliferation rate was calculated by using the following formula: number of EdU-stained cells/number of Hoechst-stained cells × 100%.

### 2.8. Cell Vitality Detection Using the Cell Counting Kit-8 (CCK-8) Assay

Cell viability was assessed using the CCK-8 Cell Counting Kit protocol (Vazyme, Nanjing, China). Relative cell viability can be indirectly assessed by measuring optical density (OD) at 450 nm using a Tecan Infinite F200/M200 multi-function microplate reader (Tecan, Männedorf, Switzerland). When myoblasts reach a confluence suitable for transfection, the OD value is used as the OD value at 0 h. Cells are then transfected, with 6–8 replicate wells per group. OD values are measured 24 h after transfection and again at 48 h and 72 h.

### 2.9. Cell Cycle Analysis via Flow Cytometry

Cells were treated according to the instructions of the Cell Cycle Detection Kit (Beyotime, Shanghai, China). First, myoblasts were plated and transfected in 6-well plates. Then, 24–48 h after transfection, the cells were detached using trypsin and collected via centrifugation into a 1.5 mL tube. Subsequently, they were resuspended in pre-cooled 70% ethanol and fixed via incubation at 4 °C for 12–16 h. Before measurement, they were stained with freshly prepared propidium iodide staining solution at 37 °C in the dark for 30 min. After PI staining, the DNA content of the cells was detected using a flow cytometer (BD, Franklin Lakes, NJ, USA), and then the DNA content distribution was assessed with FlowJo software (V 10.8.1, BD Biosciences, Franklin Lakes, NJ, USA).

### 2.10. Cell Immunofluorescence Assay

Immunofluorescence staining was used to analyze cell differentiation. Before performing the cell immunofluorescence assay, myoblasts were seeded into a 24-well plate containing complete culture medium. At 24–48 h after transfection, when the cells reached 90% confluence, the complete culture medium was replaced with differentiation medium and cultured for 5–7 days. As described in previous experiments [24], first, the cells were washed with PBS 2–3 times, 4% paraformaldehyde was added, and the cells were incubated at room temperature for 30 min to fix them. The cells were then washed three times with PBS, permeabilized with 0.5% Triton X-100 at room temperature for 20 min, washed three times with PBS, and then incubated with 5% goat serum at room temperature for 1 h. The cells were incubated with the prepared primary antibody solution (MYH3 Antibody, 1:100, Affinity Bioscience, Cincinnati, OH, USA, REF#DF9647) at 4 °C overnight. The primary antibody targets the protein MYH3. followed by three washes with PBS and incubation with the prepared secondary antibody solution (Goat Anti-Rabbit IgG H&L (Alexa Fluor^®^ 488), 1:200, Abcam, Cambridge Science Park, Cambridge, UK, REF#ab150077) at 37 °C in the dark for one hour. Finally, the cells were incubated with DAPI staining solution (Boster Biological Technology, Wuhan, China, Catalog#AR1176) at room temperature in the dark for 3–5 min, rinsed with PBS, and immediately placed under an inverted fluorescence microscope (Nikon, Minato, Tokyo, Japan) for photography.

### 2.11. Statistical Analysis

All results in this experiment were analyzed using SPSS software (IBM SPSS Statistics 19, SPSS, Inc., Chicago, IL, USA). Differences between groups were compared using an independent samples *t*-test for two groups and one-way analysis of variance (ANOVA) for multiple groups. All experimental results are presented in graphical form, with error bars representing the standard error (SEM). A *p*-value < 0.05 was considered statistically significant.

## 3. Results

### 3.1. Isolation and Purification of Fetal Sheep Myoblasts and Quantitative Identification of MRFs

**Figure 1:**
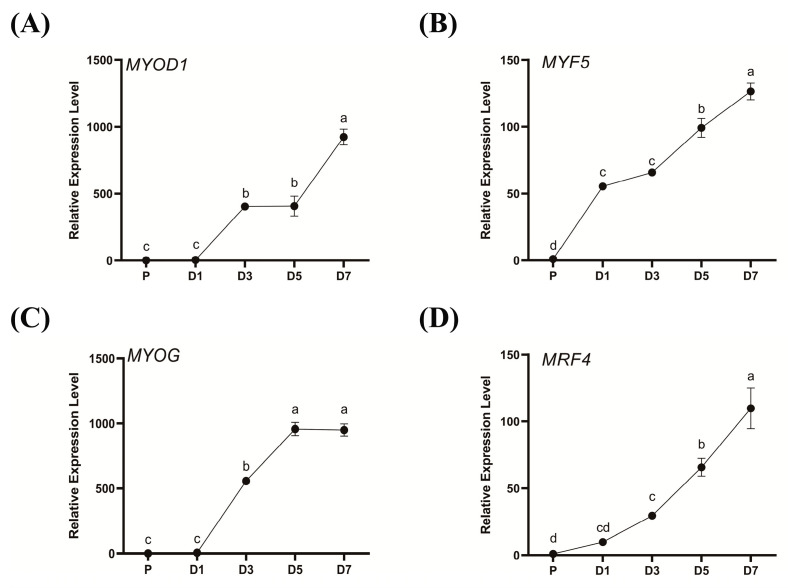
Expression analysis of MRFs in fetal sheep myoblasts at different differentiation time points (**A**–**D**): RT-PCR was used to detect the relative expression of *MyoD1* (**A**), *Myf5* (**B**), *MyoG* (**C**), and *MRF4* (**D**) in fetal sheep myoblasts on days 1, 3, 5, and 7 of differentiation induction. Different lowercase letters indicate significant differences (*p* < 0.05).

This study successfully isolated and purified fetal sheep myoblasts using the Type II collagenase digestion method. We observed that the purified fetal sheep myoblasts grew well, exhibiting a distinctly elongated spindle shape, and formed myotubes after induced differentiation (Figure S1). Subsequently, we used RT-PCR to detect the relative expression of MRFs in myoblasts at different times of differentiation induction, and took day 0 (P) without changing the differentiation medium as the control group. We found that the expression levels of *MyoD1* and *MyoG* gradually increased from day 3 to day 7 of differentiation induction (Figure 1B,D). The relative expression levels of *Myf5* and *MRF4* gradually increased during the seven days of differentiation induction and reached a peak on day 7 (Figure 1C,E) (*p* < 0.05). These results confirmed that the isolated fetal sheep myoblasts were of high purity and differentiation potential, establishing them as a reliable model for subsequent research.

### 3.2. Effect of LYPLA1 Interference on the Proliferation of Fetal Sheep Myoblasts

**Figure 2:**
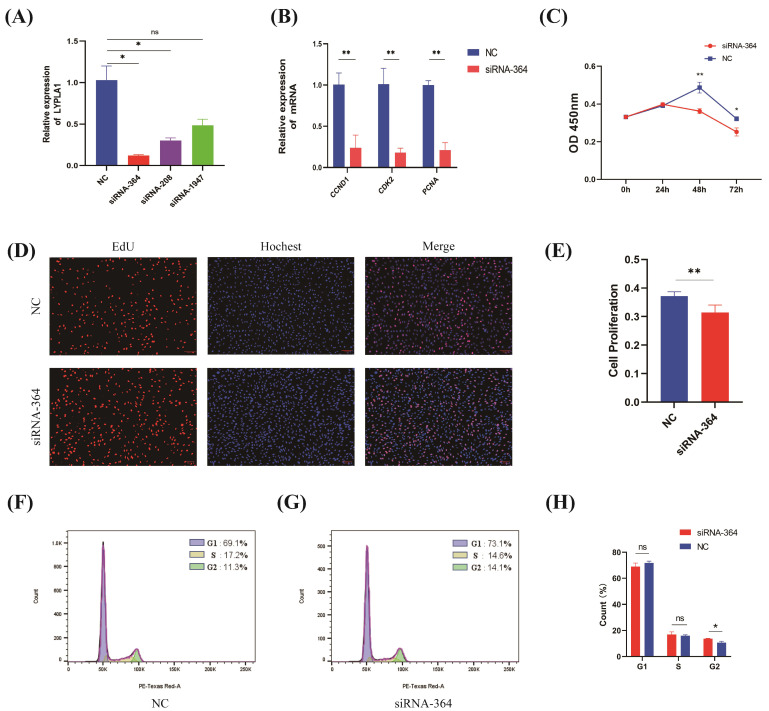
Functional verification of the effect of *LYPLA1* interference on fetal sheep myoblast proliferation. (**A**) RT-PCR detection of siRNA efficiency targeting *LYPLA1* gene expression in fetal sheep myoblasts; (**B**) RT-PCR detection of relative expression levels of proliferation marker genes in fetal sheep myoblasts transfected with siRNA-364; (**C**) detection of cell viability using the CCK-8 assay in fetal sheep myoblasts following siRNA-364 transfection at 0 h, 24 h, 48 h, and 72 h; (**D**) EdU staining image of fetal sheep myoblasts transfected with siRNA-364; (**E**) effect of siRNA-364 transfection on the proliferation rate of fetal sheep myoblasts; (**F**,**G**) flow cytometry detection of the distribution of fetal sheep myoblasts at different cell cycle stages after transfection with siRNA-364; (**H**) percentage of fetal sheep myoblasts at different cell cycle stages after transfection with siRNA-364. * *p* < 0.05; ** *p* < 0.01; ns, not significant (*p* > 0.05); n = 3.

To investigate the function of the *LYPLA1* gene, we transfected siRNA targeting *LYPLA1* into fetal sheep myoblasts. The relative expression of the *LYPLA1* gene was quantified using RT-qPCR. A significant reduction in the relative expression level of the *LYPLA1* gene was observed in the siRNA-364 and siRNA-208 treatment groups (*p* < 0.05) (Figure 2A). siRNA-364 exhibited stronger interference efficiency than siRNA-208 and was hence chosen for further analysis. We transfected siRNA-364 into fetal sheep myoblasts and performed RT-PCR, CCK-8 assays, EdU staining, and flow cytometry to analyze cell cycle distribution. RT-PCR results showed that the relative expression of proliferation marker genes in the siRNA-364-treated group was significantly lower than that in the control group (*p* < 0.01) (Figure 2B). CCK-8 assays for cell viability revealed that the OD values of the siRNA-364-treated group were significantly lower than those in the control group at both 48 and 72 h (*p* < 0.05) (Figure 2C), indicating that *LYPLA1* knockdown reduced cell viability. EdU staining revealed that the proportion of EdU-positive cells in the siRNA-364-treated group was significantly lower than that in the control group (*p* < 0.01) (Figure 2D,E). Cell cycle analysis revealed that the number of cells in the G2 phase was significantly increased in the siRNA-364-treated group compared with the control group (*p* < 0.05) (Figure 2F–H), indicating that *LYPLA1* knockdown significantly inhibited cell proliferation.

### 3.3. Effects of LYPLA1 Gene Overexpression on the Proliferation of Fetal Sheep Myoblasts

**Figure 3:**
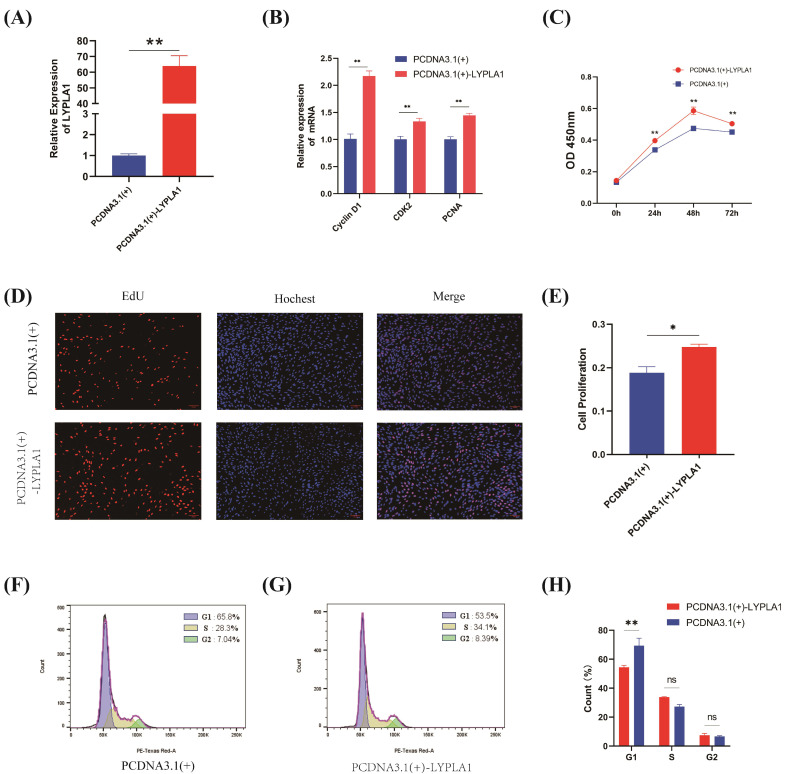
Functional validation of the effect of overexpression of *LYPLA1* on the proliferation of fetal sheep myoblasts. (**A**) RT-PCR detection of the efficiency of pcDNA3.1(+)-*LYPLA1* targeting *LYPLA1* gene expression in fetal sheep myoblasts; (**B**) RT-PCR detection of the relative expression levels of proliferation marker genes in fetal sheep myoblasts transfected with pcDNA3.1(+)-*LYPLA1*; (**C**) detection of cell viability using the CCK-8 assay in fetal sheep myoblasts following pcDNA3.1(+)-*LYPLA1* transfection at 0 h, 24 h, 48 h, and 72 h. (**D**) EdU staining image of fetal sheep myoblasts transfected with pcDNA3.1(+)-*LYPLA1*; (**E**) proliferation rate of fetal sheep myoblasts transfected with pcDNA3.1(+)-*LYPLA1*; (**F**,**G**) flow cytometry detection of the distribution of fetal sheep myoblasts at different cell cycle stages transfected with pcDNA3.1(+)-*LYPLA1*; (**H**) percentage of fetal sheep myoblasts at different cell cycle stages transfected with pcDNA3.1(+)-*LYPLA1*. * *p* < 0.05; ** *p* < 0.01; ns, not significant (*p* > 0.05); n = 3.

To investigate whether *LYPLA1* overexpression also affects the proliferation of fetal sheep myoblasts, in this study, a recombinant *LYPLA1* overexpression plasmid was successfully constructed. We transfected the overexpression recombinant plasmid into fetal sheep myoblasts and assayed the relative expression of *LYPLA1* using RT-PCR. It was observed that the relative expression of *LYPLA1* in the pcDNA3.1(+)-*LYPLA1*-treated group was significantly higher than that in the control group (*p* < 0.01) (Figure 3A). Subsequently, RT-PCR was used to assay the relative expression of proliferation marker genes, which was significantly higher in the pcDNA3.1(+)-*LYPLA1*-treated group than in the control group (*p* < 0.01) (Figure 3B). Cell viability assays using CCK-8 assays showed that the OD values of the pcDNA3.1(+)-*LYPLA1*-treated group were significantly higher than those in the control group at 24, 48, and 72 h (*p* < 0.01) (Figure 3C). EdU staining results showed that the proportion of EdU-positive cells in the pcDNA3.1(+)-*LYPLA1*-treated group was significantly higher than that in the control group (*p* < 0.05) (Figure 3D,E), indicating that increased *LYPLA1* gene expression enhances cell viability. Cell cycle analysis revealed a significant increase in the number of cells in the G1 phase in the control group compared with the treated group (*p* < 0.01) (Figure 3F–H), indicating that cell proliferation was arrested in the control group. These results suggest that changes in *LYPLA1* gene expression significantly affect the proliferation of fetal sheep myoblasts.

### 3.4. Effects of LYPLA1 Gene Interference on the Differentiation of Fetal Sheep Myoblasts

**Figure 4:**
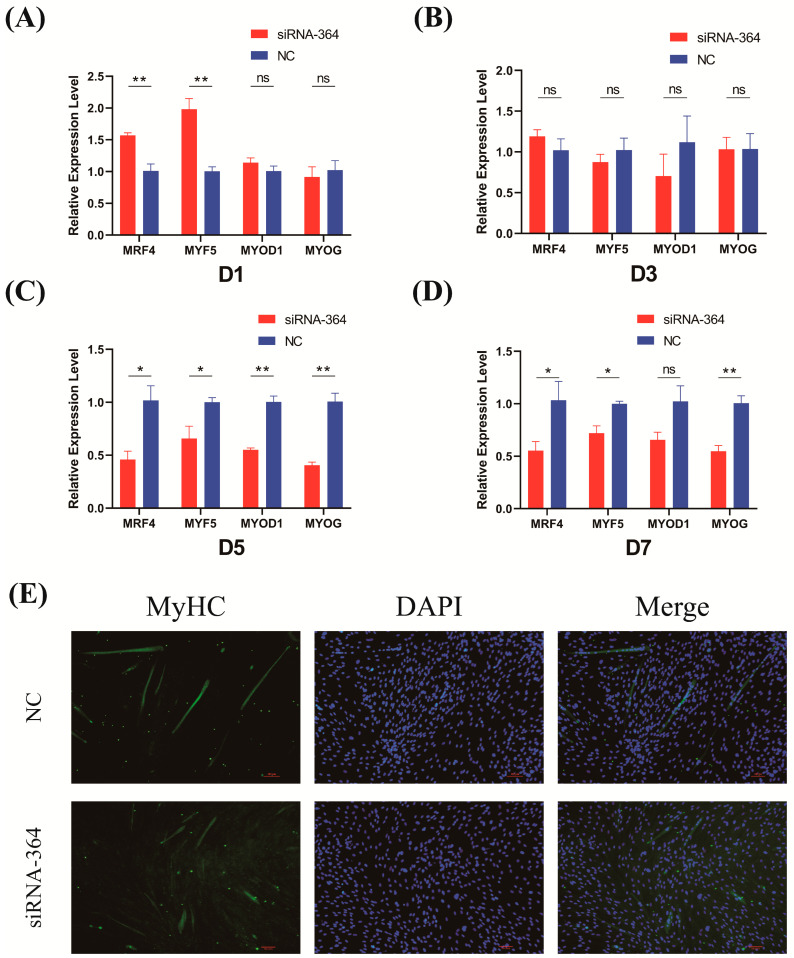
Functional verification of the effect of *LYPLA1* gene interference on fetal sheep myoblast differentiation. (**A**–**D**) RT-PCR detection of the relative expression levels of *MyoD1*, *Myf5*, *MyoG*, and *MRF4* on days 1, 3, 5, and 7 of induction differentiation of fetal sheep myoblasts transfected with siRNA-364; (**E**) inverted fluorescence microscopy observation of MyHC immunofluorescence staining on day 7 of induction differentiation of fetal sheep myoblasts transfected with siRNA-364. * *p* < 0.05; ** *p* < 0.01; ns, not significant (*p* > 0.05); n = 3.

To further investigate the effect of *LYPLA1* gene expression on fetal sheep myoblast differentiation, we transfected fetal sheep myoblasts with siRNA-364 and replaced the growth medium with differentiation medium to establish an in vitro differentiation model. Total RNA was extracted from cells on days 1, 3, 5, and 7 of differentiation induction. Relative expression of *MyoD1*, *MyoG*, *Myf5*, and *MRF4* was assessed by RT-PCR using reverse-transcribed cDNA as a template. RT-PCR results showed that *LYPLA1* knockdown upregulated the relative expression of *Myf5* and *MRF4* on days 1–3 of in vitro differentiation (*p* < 0.01) (Figure 4A,B). However, on days 5–7 of in vitro differentiation induction, *LYPLA1* knockdown significantly downregulated the mRNA levels of key myogenic regulatory factors (*MyoD1*, *MyoG*, *Myf5*, and *MRF4*) (*p* < 0.05 or *p* < 0.01) (Figure 4C,D). Furthermore, indirect immunofluorescence analysis of MyHC on day 7 of differentiation revealed clear inhibition of myogenesis in the siRNA-364-treated group versus the control, with fewer fused myotubes and a lower degree of differentiation. These results indicate that *LYPLA1* gene knockdown inhibits late-stage myoblast differentiation.

### 3.5. Effects of LYPLA1 Gene Overexpression on the Differentiation of Fetal Sheep Myoblasts

**Figure 5:**
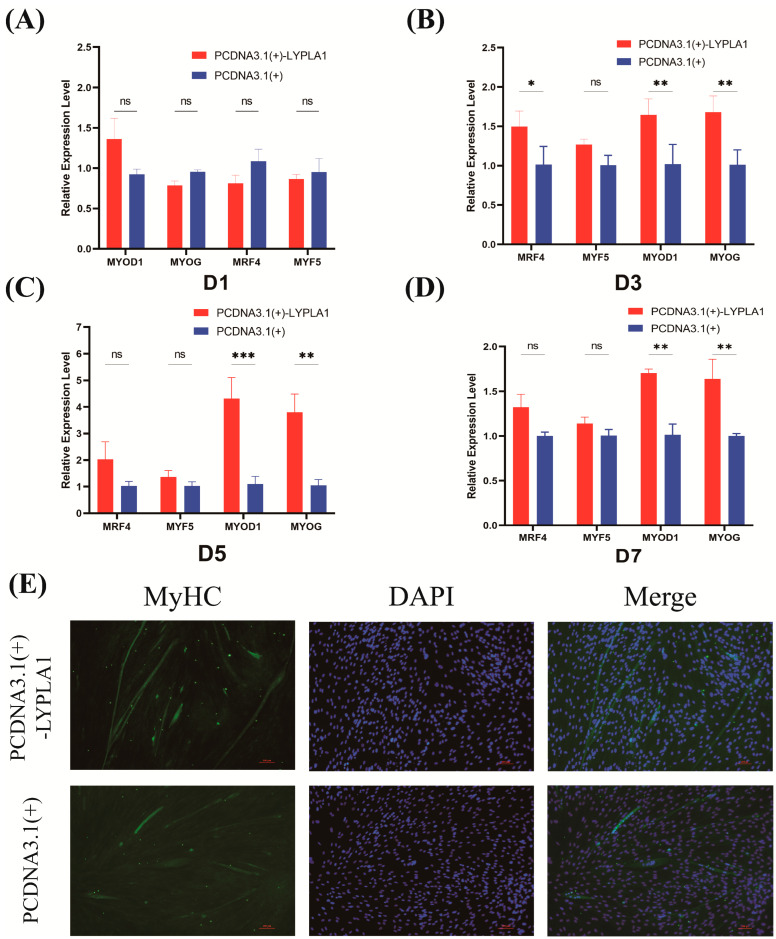
Functional verification of the effect of *LYPLA1* gene overexpression on fetal sheep myoblast differentiation. (**A**–**D**) RT-PCR detection of the relative expression levels of *MyoD1*, *Myf5*, *MyoG*, and *MRF4* in fetal sheep myoblasts transfected with pcDNA3.1(+)-*LYPLA1* on days 1, 3, 5, and 7 of induction differentiation; (**E**) inverted fluorescence microscopy observation of MyHC immunofluorescence staining in fetal sheep myoblasts transfected with pcDNA3.1(+)-*LYPLA1* on day 7 of induction differentiation. * *p* < 0.05, ** *p* < 0.01, *** *p* < 0.001, ns *p* > 0.05, n = 3 biological replicates.

Then, we transfected the *LYPLA1* gene overexpression recombinant plasmid into fetal sheep myoblasts to explore whether its overexpression also affects their differentiation. RT-PCR analysis was performed to profile the expression of key myogenic regulatory factors (*MyoD1*, *MyoG*, *Myf5*, and *MRF4*) on days 1, 3, 5, and 7 of differentiation. RT-PCR results showed that *LYPLA1* overexpression significantly upregulated the mRNA levels of key myogenic regulatory factors (*MyoD1*, *MyoG*, *Myf5*, and *MRF4*) on days 1–7 of differentiation (Figure 5A–D). Indirect immunofluorescence analysis of MyHC on day 7 of myoblast differentiation (Figure 5E) showed that myogenic differentiation was significantly promoted in the pcDNA3.1(+)-*LYPLA1*-treated group compared with the control group, with a higher number of fused myotubes and a higher degree of differentiation. Therefore, *LYPLA1* overexpression promotes myoblast differentiation. In summary, changes in *LYPLA1* gene expression can significantly affect the differentiation process of fetal sheep myoblasts.

## 4. Discussion

The protein encoded by the *LYPLA1* gene exhibits depalmitoylation and lysophospholipase activity [25,26] and may be involved in Ras localization and signal transduction [9]. Its expression is broadly distributed across tissues such as the muscle, lens, and kidney [27]. GWAS has provided evidence that the *LYPLA1* gene is a candidate gene affecting birth weight and weaning weight [28]. Based on this, we speculate that *LYPLA1* may regulate the proliferation and differentiation of myoblasts at the transcriptional and translational levels by modulating the palmitoylation cycle of key proteins (such as Ras itself) in the Ras signaling pathway.

In this study, fetal sheep myoblasts were successfully isolated and purified, and an in vitro differentiation model was established. Quantitative analysis demonstrated a progressive increase in *MyoD1* and *MyoG* expression levels from day 3 to day 7 of differentiation. In addition, the relative expression levels of *Myf5* and *MRF4* reached a peak on the seventh day of differentiation induction, and the changes in MRF expression in this study were consistent with the reported expression pattern [29]. Experiments have shown that the activation of *Myf5* and *MyoD1* during myoblast proliferation is a determinant of early myogenesis, and *MyoG* and *MRF4* act downstream to strictly control and maintain terminal differentiation [30,31,32]. Therefore, the myoblasts extracted in this experiment have high purity and good differentiation potential, which can provide a reliable in vitro model for subsequent functional studies of the *LYPLA1* gene.

To elucidate the role of *LYPLA1* in myoblast proliferation, we performed both gain- and loss-of-function studies. The results of this experiment showed that the *LYPLA1* gene is a positive regulator of myoblast proliferation. After interfering with the expression of the *LYPLA1* gene, cell viability and proliferation rate were significantly decreased, and the cell cycle was arrested in the G2 phase. In contrast, overexpression of the *LYPLA1* gene significantly promoted cell viability and proliferation rate. This finding is consistent with the reported function of the *LYPLA1* gene in cell cycle regulation and growth in other cell types. For example, Zhang et al. found in a study on cervical cancer that overexpression of the *LYPLA1* gene can promote cervical cancer cells to overcome cell cycle arrest and promote proliferation [33]. Similar results were found in another study on non-small cell lung cancer [34]. In this regard, we believe that the *LYPLA1* gene may play an important role in different cells and different biological processes.

Evidence indicates that myogenic differentiation requires prior cell cycle exit, defining proliferation and differentiation as distinct biological phases [35,36]. To further investigate the impact of *LYPLA1* on myoblast differentiation, we knocked down *LYPLA1* expression in an in vitro differentiation model. We found that knockdown of *LYPLA1* slightly upregulated some MRFs (such as *Myf5* and *MRF4*) during the early stages of differentiation (days 1–3) but significantly inhibited the expression of all key MRFs and myotube formation during the critical mid to late stages of differentiation (days 5–7). We hypothesize that this phenomenon may reflect an early compensatory mechanism activated by cells in response to the loss of *LYPLA1* function. Since *LYPLA1* is involved in regulating cell cycle exit, its knockdown may attempt to initiate differentiation by temporarily upregulating the expression of certain early myogenic regulatory factors. However, this initial gene expression compensation failed to effectively advance the differentiation process. Furthermore, overexpression of *LYPLA1* induced differentiation and strongly upregulated MRF expression throughout the entire differentiation phase and significantly promoted myotube formation. These results suggest that insufficient *LYPLA1* expression may prevent myoblasts from successfully transitioning from proliferation to differentiation, resulting in severe defects in the late stages of differentiation. Overall, we found that changes in *LYPLA1* expression affect the differentiation process of ovine embryonic myoblasts. These findings provide important theoretical support for the potential application of *LYPLA1* as a molecular marker for ovine breeding and improvement.

In summary, this study, based on the growth and development of sheep muscle, revealed for the first time that the *LYPLA1* gene promotes proliferation and differentiation in fetal sheep myoblasts. These results will provide an important theoretical basis for the use of the *LYPLA1* gene as a potential molecular marker for sheep breeding improvement.

## 5. Conclusions

Finally, this study revealed the role of the *LYPLA1* gene in regulating the proliferation and differentiation of fetal sheep myoblasts. Through this study, we hope to provide an important theoretical basis for the use of the *LYPLA1* gene as a potential molecular marker for improving meat traits and also to open up new ideas for its further application in sheep molecular breeding in the future.

## Figures and Tables

**Table 1:** Sequence information for siRNA and NC.

Gene Name	siRNA Name	Sequence (5′-3′)
*LYPLA1*	siRNA-208	F: CCAUCAUGGUUUGACAUUATT R: UAAUGUCAAACCAUGAUGGTT
	siRNA-364	F: GGAGCUUUAUCUCUGUACATT R: UGUACAGAGAUAAAGCUCCTT
	siRNA-1947	F: AUUUUUCAAACUAUCGGUUTT R: AACCGAUAGUUUGAAAAAUTT
NC	NC-F	UUCUCCGAACGUGUCACGUTT
	NC-R	ACGUGACACGUUCGGAGAATT

**Table 2:** Primers used in plasmid construction.

Gene Name	Primer Name	Sequence (5′-3′)	Product Length
*LYPLA1*	*LYPLA1*-F	ggagacccaagctggctagcTGTATGTGCGGCAATAACATGTC	679 bp
	*LYPLA1*-R	aacgggccctctagactcgagGACGATGCTGGTGTGCACTTC	

Note: In the primer sequences, the lowercase letters at the 5′-end represent the 15 bp homologous arm from the linearized pcDNA3.1(+) vector. The underlined lowercase letters denote the restriction enzyme sites. The remaining uppercase letters correspond to the insertion fragment sequences.

**Table 3:** Specific primers used for RT-PCR.

Gene Name	Primer Name	Sequence (5′-3′)	GenBank Accession
*LYPLA1*	*LYPLA1*-F	GATTGGGAGACACAGGGCAT	NM_001306103.1
	*LYPLA1*-R	GCATGCGGGCAGATGTATTT	
*CDK2*	*CDK2*-F	TGGGCCAGGCAGGATTTTAG	NM_001142509.1
	*CDK2*-R	GTCGAAGGTGAGGTACTGGC	
*Cyclin D1*	*Cyclin D1*-F	GCTTCCTCTCCTATCACCGC	XM_027959928.2
	*Cyclin D1*-R	GGCTTTGGGGTCCAAGTTCT	
*PCNA*	*PCNA*-F	TCTGCAAGTGGAGAACTTGGAA	XM_004014340.5
	*PCNA*-R	AGGAGACAGTGGAGTGGCTT	
*MyoD1*	*MyoD1*-F	AACTGTTCCGACGGCATGAT	NM_001009390.1
	*MyoD1*-R	TGTAGTAAGCGCGGTCGTAG	
*Myf5*	*Myf5*-F	CCTCAAGTTGCTCTGATGGC	XM_015094556.3
	*Myf5*-R	ATCCAGGTTGCTCTGAGTTGG	
*MyoG*	*MyoG*-F	CTCAACCAGGAGGAGCGTGAT	NM_001174109.1
	*MyoG*-R	GTGGGCATCTGTAGGGTCCG	
*MRF4*	*MRF4*-F	GCTACAGACCCAAGCAGGAA	NM_001134782.1
	*MRF4*-R	CGAGGCCGATGAATCAATGC	
*GAPDH*	*GAPDH*-F	TCTCAAGGGCATTCTAGGCTAC	NM_001190390.1
	*GAPDH*-R	GCCGAATTCATTGTCGTACCAG	

## Data Availability

The original contributions presented in this study are included in the article/Supplementary Material. Further inquiries can be directed to the corresponding author.

## Supplementary Materials

The following supporting information can be downloaded at https://www.mdpi.com/article/10.3390/ani15233474/s1. Figure S1: Morphology of fetal sheep myoblasts during induction of differentiation 1–7 days.
